# Elevated maternal testosterone induces sex-specific neurodevelopmental changes and ASD-related behavioral phenotypes in rat offspring

**DOI:** 10.1038/s41390-025-04425-y

**Published:** 2025-10-01

**Authors:** Jay S. Mishra, Sai Krishna Bhamidipati, Jordan Ronald Ross, Sri Vidya Dangudubiyyam, Jayshree Samanta, Sathish Kumar

**Affiliations:** 1 Department of Comparative Biosciences, School of Veterinary Medicine, University of Wisconsin-Madison, Madison, WI USA; 2 Department of Obstetrics and Gynecology, School of Medicine and Public Health, University of Wisconsin-Madison, Madison, WI USA; 3 Present Address: Department of Biomedical Sciences, College of Veterinary Medicine, University of Georgia, Athens, GA USA

## Abstract

**Objective:**

Elevated maternal testosterone (T) during pregnancy disrupts neurodevelopment and behavior in offspring, mimicking features of autism spectrum disorder (ASD).

**Methods:**

In a rat study, dams received daily T injections (0.5 mg/kg) from gestational days 12–20, doubling maternal plasma T to mimic levels seen in pregnancy complications. Controls received vehicle. Offspring were assessed neonatally (postnatal day 9) for communication (ultrasonic vocalizations), neurogenesis (NeuN+ neurons), myelination (MBP+ area), and brain docosahexaenoic acid (DHA). Adolescent offspring (6–8 weeks) underwent behavioral tests for cognition (Y-maze, novel object recognition) and sociability (three-chamber test).

**Results:**

T-exposed pups had lower birth weights and reduced vocalizations during maternal separation. Sex-specific neural changes observed: males showed reduced cortical neuron density, while females had diminished corpus callosum myelination. Both sexes exhibited decreased brain DHA. In adolescence, T offspring displayed cognitive deficits (impaired spatial/recognition memory) and social impairments (reduced sociability and social novelty preference).

**Conclusion:**

The study highlights maternal T as a risk factor for neurodevelopmental disorders, with sex-specific effects on brain structure and function. Reduced brain DHA suggests a mechanistic link, implicating lipid metabolism in T-associated neurodevelopmental disruptions. These findings support further exploration of DHA supplementation as a therapeutic strategy to mitigate adverse outcomes in high-risk pregnancies.

**Impact:**

- Elevated maternal testosterone (T) during pregnancy induces ASD-like neurobehavioral deficits (e.g., impaired communication, social/cognitive dysfunction) and sex-specific neural alterations in offspring.
- Prenatal T differentially impacts male vs. female brain structure: T-exposed males show cortical neuron loss, while females exhibit myelination deficits in the corpus callosum.
- First to connect maternal T-driven offspring brain docosahexaenoic acid (DHA) reduction to neurodevelopmental impairment.
- Supports prenatal DHA supplementation as a strategy to mitigate neurodevelopmental risks in high-T pregnancies.
- Informs policies addressing rising neurodevelopmental disorder rates linked to maternal metabolic/endocrine imbalances.

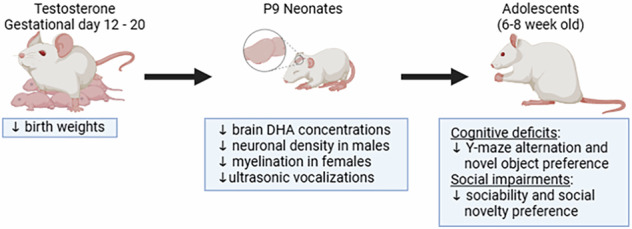

## Introduction

The etiology of psychiatric disorders is increasingly understood as a complex interplay between genetic predisposition and early-life environmental exposures, particularly during critical windows of fetal development.^1^ Among these exposures, dysregulation of maternal sex hormones has emerged as a significant contributor to neurodevelopmental disruptions. Epidemiological and preclinical studies link hormonal imbalances, such as elevated maternal testosterone (T), to fetal growth restriction and long-term behavioral deficits in offspring.^2–5^ As a key sex hormone, T plays a profound role in shaping mammalian brain development, influencing neural circuitry that governs social behavior, cognition, and emotional regulation.^6^ Clinical studies highlight this relationship, reporting correlations between elevated maternal T levels and increased autism spectrum disorder (ASD) traits in children, particularly male offspring.^7–9^ Notably, maternal polycystic ovary syndrome (PCOS)—a hyperandrogenic condition—is also associated with a higher prevalence of ASD diagnoses, but predominantly in female offspring.^10,11^ Preclinical models further strengthen this link, demonstrating that prenatal T exposure alters dendritic spine density and morphology in offspring, structural changes that mirror observations in individuals with ASD and attention-deficit/hyperactivity disorder (ADHD).^12,13^ Together, these findings underscore the need to investigate how maternal T influences neurodevelopmental trajectories, particularly through mechanisms bridging maternal hormonal imbalance and fetal neural dysfunction.

Docosahexaenoic acid (DHA), a long-chain polyunsaturated fatty acid (PUFA) constituting ~30% of brain cell membranes, is indispensable for neurodevelopment due to its roles in neuronal membrane fluidity, synaptogenesis, and neuroinflammation regulation.^14–16^ During late gestation—a critical period marked by neuronal migration, synaptogenesis, and myelination^17,18^ —both human and rodent fetuses rely entirely on placental transfer of maternal DHA to support rapid brain growth.^19^ Postnatally, DHA levels surge 30-fold within the first two years of human life, reflecting its ongoing importance for neural connectivity and cognitive maturation.^20,21^ Maternal PUFA supplementation during pregnancy not only improves infant DHA status^22,23^ but also enhances neurodevelopmental outcomes, including language acquisition and executive function.^24–26^ Conversely, prenatal DHA deficiency disrupts neurogenesis,^27^ reduces neuron size in regions like the prefrontal cortex and hippocampus,^28^ and lowers serotonin and dopamine concentrations.^29^ Lower PUFAs, particularly DHA, have been observed in individuals with ASD^30–32^ and linked with cognitive and behavioral impairments, memory dysfunction, and neurodegenerative diseases.^33–35^ Importantly, maternal conditions such as preeclampsia and gestational diabetes—which dysregulate placental DHA metabolism—result in reduced fetal DHA levels.^36–39^ Children born under these conditions exhibit lifelong neurocognitive impairments, including higher rates of ASD, ADHD, and lower intelligence quotients^40–42^ underscoring DHA’s pivotal role in linking maternal health and offspring neurodevelopment.

A critical gap remains in understanding how maternal T exposure intersects with fetal DHA availability to influence neurodevelopment. While experimental studies suggest maternal T reduces fetal plasma DHA levels,^43^ its direct effects on fetal brain DHA concentrations—and downstream consequences for neurogenesis, myelination, and long-term neurobehavioral outcomes—remain unknown. To address this gap, we investigated the impact of elevated maternal T across two developmental stages: (a) Neonatal outcomes: Brain DHA levels, neurogenesis (cortical neuronal counts), myelination (corpus callosum integrity), and ultrasonic vocalizations—a validated measure of early communicative behavior in rodents. (b) Adolescent outcomes: Cognitive function (spatial working memory, novel object recognition) and social behavior (sociability, social novelty preference), with emphasis on sex-specific effects to clarify disparities in neurodevelopmental susceptibility.

## Methods

### Experimental animals and treatment

Timed-pregnant Sprague-Dawley rats (gestational day [GD] 8) were obtained from Charles River Laboratories (Wilmington, MA) and housed in AAALAC-accredited facilities under a 12 h light/dark cycle with ad libitum access to food and water. From GD 12 to GD 20, dams received daily subcutaneous injections of T propionate (0.5 mg/kg) dissolved in sesame oil, a dose previously shown to elevate maternal plasma T levels twofold, mimicking concentrations observed in complicated human pregnancies.^44,45^ Control dams received vehicle (sesame oil) injections. Maternal weight and plasma T levels were measured at GD 20. Pups were delivered vaginally, and birth weights were recorded on postnatal day (P) 0. On P9, offspring underwent assessments of ultrasonic vocalizations, neurogenesis, myelination, and brain DHA concentration. In adolescent offspring (6–8 weeks of age), the behavioral tests, including spatial working memory (spontaneous alternation in the Y-maze), recognition memory (novel object recognition task), and social behavior (Three-chamber sociability and social novelty preference tests) were conducted. All procedures were approved by the University of Wisconsin-Madison Institutional Animal Care and Use Committee (IACUC protocol #V005847) and adhered to the NIH *Guide for the Care and Use of Laboratory Animals*.

### Plasma T quantification

Plasma T levels were quantified using a commercially available ELISA kit (Enzo Life Sciences, Farmingdale, NY) following established protocols from our previous studies.^46,47^ The assay sensitivity was 6 pg/mL, with intra- and inter-assay coefficients of variation (CV) < 5%.

### Ultrasonic vocalization

Pups were isolated from their mothers and individually placed in a sound-attenuated isolation chamber (white plastic walls) for recording. An ultrasound microphone (Avisoft Bioacoustics, Nordbahn, Germany) was positioned 10 cm above the chamber floor. Ultrasonic vocalizations were recorded for 5 min using an Avisoft UltraSoundGate 116 USB acquisition device and Avisoft Recorder software (sampling rate: 300 kHz, 16-bit resolution). Following recording, pups were marked with a non-toxic surgical marker and returned to their home cage. The chamber was cleaned with 70% ethanol and allowed to dry between trials to eliminate residual odors. For analysis, the first 90 s of each recording were processed using Avisoft-SASLab-Pro software. A high-pass filter (25 kHz cutoff) was applied to remove background noise,^48,49^ and vocalizations were quantified based on total call numbers.

### Immunofluorescence

Postnatal day 9 pups were transcardially perfused with cold phosphate-buffered saline (PBS) followed by 4% paraformaldehyde (PFA). Brains were immersion post-fixed in 4% PFA for 2–4 h at 4 °C, cryoprotected by incubating overnight in 30% sucrose on a shaker at 4 °C, followed by embedding in optimal cutting temperature compound for cryostorage at −80 °C. The cryopreserved brains were sectioned on a Leica CM3050S cryostat to obtain 20 μm coronal sections starting from the rostral end of the corpus callosum (forceps minor) to the anterior hippocampus caudally (bregma 2.7 mm to −1.4 mm). For immunofluorescence, we used 10 sections/brain with 200 μm distance between sections, sampling the entire rostrocaudal span of the forebrain corpus callosum. Sections were fixed in cold methanol for 10 minutes, blocked for 1 hour at room temperature in PBS containing 10% normal goat serum, 0.1% bovine serum albumin, and 0.3% Triton X-100. Sections were then incubated overnight at 4 °C with mouse anti-MBP (myelin basic protein; 1:200, MilliporeSigma, Burlington, MA; Cat# MAB386) or anti-NeuN (neuronal nuclei; 1:200, MilliporeSigma, Burlington, MA; Cat# MAB377) primary antibodies. After PBS washes, sections were incubated for 1 h at room temperature with goat anti-mouse Alexa Fluor 488- or 568-conjugated secondary antibodies (1:1000, Invitrogen, ThermoFisher Scientific, Newark, DE; Cat# A-11034, A-11036) and counterstained with Hoechst 33342 (1:5000, Invitrogen, ThermoFisher Scientific, Newark, DE; Cat# H3570) to label nuclei.

Epifluorescent images were obtained as Z-stacks of 5 µm optical sections using a Keyence BZX700 microscope with inbuilt automatic deconvolution at 20× magnification. For quantification of cortical neurons, images were acquired from two fields per hemisphere (total four fields per section): one from the anterior cingulate cortex (ACC; medial prefrontal cortex) and one from the sensorimotor cortex (lateral frontal lobe). NeuN+ cells and Hoechst-stained nuclei were manually counted using the cell counter plugin in Fiji ImageJ. Data from the ACC and sensorimotor cortex were pooled as ‘frontal cortex’ due to comparable neuronal density (p > 0.05) and shared axonal projections to the corpus callosum,^50,51^ enabling integrated analysis of neuron-myelin relationships. The percentage of NeuN+ cells was calculated as [(NeuN+Hoechst+ cells)/(Hoechst+ nuclei)] × 100. Counts from four fields/section, 10 sections/brain, and six brains/group were averaged per litter.

For MBP+ area quantification, tiled Z-stack images of the corpus callosum (genu and body between cingula) were acquired from 10 sections/brain and six brains/group. Images were converted to 8-bit grayscale, thresholded uniformly to isolate MBP+ areas, and the corpus callosum was manually outlined (polygon tool). The % MBP+ area within the selection was calculated and averaged per group. MBP analysis focused on the corpus callosum due to its peak postnatal myelination at P9.^52^ Publication-quality confocal images (1 μm Z-stacks; Leica TCS SP8) were processed in Adobe. Investigators were blinded to treatment groups during all procedures.

### DHA quantification using LC-MS/MS

Brain tissues were homogenized in 0.5 mL of ice-cold methanol containing deuterated DHA (DHA-d5) as an internal standard, followed by protein precipitation for 12 h at 4 °C. Lipids were extracted using C18 solid-phase extraction columns preconditioned with methanol and water, and methyl formate eluates were analyzed via liquid chromatography-tandem mass spectrometry (LC-MS/MS). Chromatographic separation was performed on a Shim-Pack GIST-HP C18 column (150 × 2.1 mm, 3 μm) with a guard column (10 × 1.5 mm, 3 μm) using an isocratic mobile phase of 90% acetonitrile, 10% water, and 2 mM ammonium acetate at a flow rate of 0.21 mL/min. The column oven and autosampler temperatures were maintained at 40 °C and 5 °C, respectively, with a 10 μL injection volume. The LCMS-8045 triple quadrupole mass spectrometer operated in negative electrospray ionization mode with an interface voltage of 3 kV, desolvation line temperature of 250 °C, heat block temperature of 400 °C, and source temperature of 300 °C. Nebulizing and drying gas (nitrogen) flows were set to 3 L/min and 10 L/min, respectively, with argon collision gas at 230 kPa. Data acquisition and processing were performed using LabSolution software (v5.91/2017), where raw spectra were smoothed using a 20-second window and integrated via the i-PeakFinder algorithm (1-degree baseline offset). Peaks were identified by absolute retention time ( ± 2% tolerance) and quantified using a 5-point calibration curve (linear regression, no weighting), with quantifier-to-qualifier ion ratios validated within ±50% tolerance.

### Y-Maze spontaneous alternation

The Y-maze apparatus (Maze Engineers, Skokie, IL) consisted of three identical arms (11 cm wide × 50 cm long) surrounded by distal spatial cues (e.g., geometric patterns, contrasting visual markers). Rats were placed in the center of the maze and allowed to explore freely for 10 minutes. An arm entry was defined as all four paws crossing into an arm. Triplets were counted only after the first complete entry into all three arms. Spontaneous alternation percentage was calculated as: Alternation (%) = (Number of Non-Repeating Arm Triplets divided by Total Arm Entries) ×100. Testing and analysis were conducted by an experimenter blinded to treatment conditions to minimize bias.

### Novel object recognition

The novel object recognition test was conducted to assess long-term object memory in rats using a gray open-field arena (60 × 60 × 40 cm; Maze Engineers, Skokie, IL). Rats underwent habituation in the empty arena for 5 minutes/day over two consecutive days to reduce stress and acclimate to the environment. On day 3 (training phase), two identical objects (size-matched glass or plastic jars; similar texture, ~15 cm height) were placed symmetrically in the arena, and rats explored them for 5 minutes. After a 48 h retention interval, one object was replaced with a novel object (distinct shape, similar size/texture), and rats were reintroduced to the arena for a 5-minute test phase. Objects and arena were cleaned with 70% ethanol between trials to eliminate odor cues. Object positions (left/right for novel vs. familiar) were counterbalanced across subjects. Experimenters wore gloves to prevent scent transfer. Exploration (sniffing or touching objects with the nose within 2 cm) was video recorded. Rats failing to explore both objects or with <10 seconds of total exploration were excluded. The recognition index was calculated as [(time with novel object) / (total exploration time)] × 100, with >50% indicating novelty preference. Video recordings from the 5-minute habituation sessions (Days 1–2) were analyzed to assess locomotor function and anxiety-like behavior. Total distance traveled (m) was quantified to rule out motor deficits. Anxiety-like behavior was evaluated by measuring time spent in the center zone and the number of center entries, defined as all four paws crossing into the zone. Analysis was performed using ANY-maze software (Stoelting Co., Wood Dale, IL).

### Three-chamber sociability and social novelty test

The 3-chamber sociability and social novelty recognition test was conducted in a rectangular gray arena (80 × 40.5 × 40 cm) divided into three equally sized chambers with transparent partitions (Maze Engineers, Skokie, IL). Two identical wire cylinder cages (15 cm diameter × 30 cm height), positioned centrally in each end chamber to minimize wall-hugging behavior, allowed auditory, visual, and olfactory interaction. The arena and cylinders were cleaned with 70% ethanol and odor-neutral detergent between trials. Rats underwent habituation (Day 1: 10 min free exploration of the empty arena), followed 24 hours later by the sociability test, where an unfamiliar, age/sex-matched rat (Stranger 1) was confined to one cylinder while the opposite chamber contained an empty cylinder. The test rat was released into the center chamber, and doorways were opened to allow 7 minutes of free exploration. Interactions (sniffing, proximity ≤2 cm, or directed attention) were analyzed from video recordings by a blinded observer using ANY-maze software (Stoelting Co., Wood Dale, IL). Sociability was calculated as Social Preference Index= [(Time with Stranger 1 − Empty)/(Total time) × 100]. Immediately following the sociability test, a social novelty phase was conducted: the empty cage was replaced with a second novel stranger rat (unfamiliar), while the original stimulus rat (now familiar) remained in its cage. The test rat explored the apparatus for 7 min, and social novelty preference was calculated as the interaction time spent with the novel stranger versus the familiar rat.

### Statistical analysis

All statistical analyses were performed using GraphPad Prism. Data are presented as mean ± SD. To account for litter effects, measurements from multiple offspring of the same dam were averaged, and the dam (n) was treated as the experimental unit. Normality (Shapiro-Wilk test) and homogeneity of variance (Brown-Forsythe test) were confirmed prior to parametric testing. Group comparisons were analyzed using two-way ANOVA (factors: treatment and sex) followed by Tukey’s post hoc test for multiple comparisons. For single comparisons between control and T groups, unpaired Student’s t-tests were used. Statistical significance was set at *p* < 0.05.

## Results

### Maternal and offspring outcomes

**Fig. 1:**
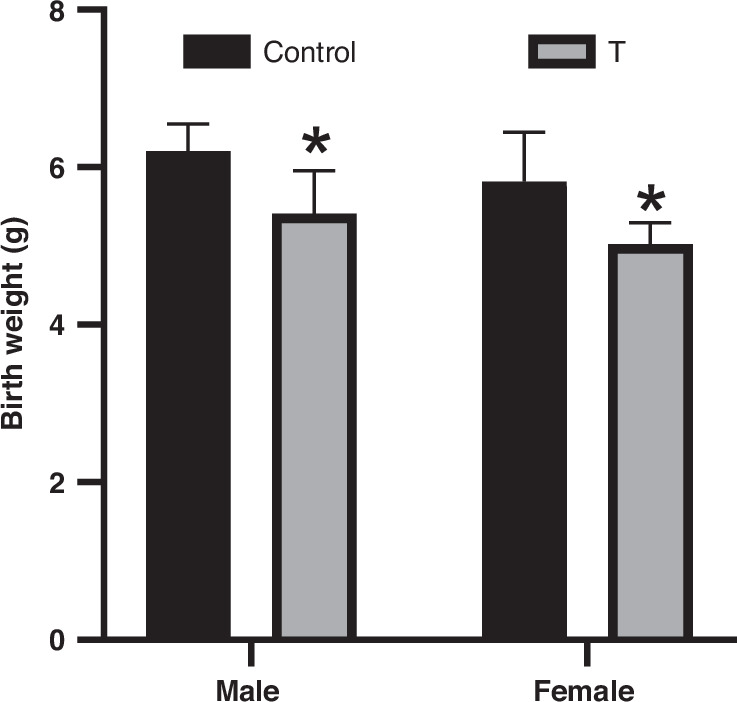
Prenatal T exposure reduces birth weight in male and female offspring. Birth weights of male and female offspring from control and T-exposed dams. Data represent mean ± SD (*n* = 6 litters/group; measurements averaged per litter). *p < 0.05 vs. sex-matched controls (two-way ANOVA with Tukey’s post hoc test).

Maternal body weight on GD 20 did not differ between control and T dams (control: 319 ± 32.5 g; T: 305 ± 29.6 g). However, T dams exhibited elevated plasma T levels (2.49 ± 0.26 ng/mL vs. 1.12 ± 0.15 ng/mL in controls; *p* < 0.001). Consistent with this hormonal difference, offspring born to T dams showed reduced birth weights (main effect of treatment: F(1,20) = 16.89, *p* = 0.0005), with T males and females exhibiting 12.7% (p = 0.0399) and 13.6% (*p* = 0.0404) reductions, respectively, compared to sex-matched controls (Fig. 1). In contrast, litter sizes (control: 12.1 ± 1.9; T: 11.7 ± 2.6) and sex ratios (percent males per litter – control: 48 ± 4.1%; T: 50 ± 3.9%) were unaffected by treatment.

### Juvenile communication deficits

**Fig. 2:**
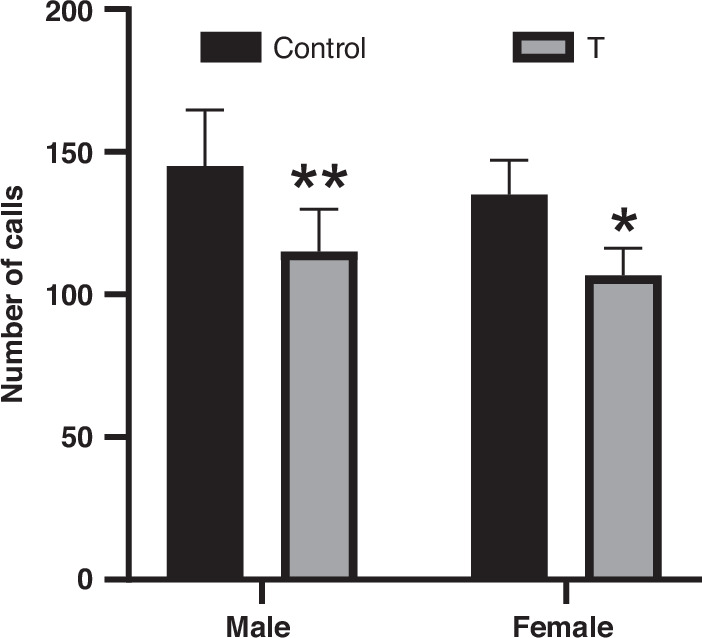
Prenatal T exposure impairs neonatal communication in male and female offspring. Total ultrasonic vocalizations (USVs) emitted by postnatal day 9 (P9) male and female offspring during a 90-second maternal separation test. Data represent mean ± SD (n = 6 litters/group). *p < 0.05, **p < 0.01 vs. sex-matched controls (two-way ANOVA with Tukey’s post hoc test).

Because traditional cognitive, social, and affective behavioral assessments are unreliable in pre-weaning rats, we quantified distress calls (ultrasonic vocalizations) elicited during maternal separation as a proxy for early juvenile communication.^53–55^ Using this approach, we found that T offspring emitted fewer ultrasonic vocalizations than controls (main effect of treatment: F(1,20) = 24.06, *p* < 0.0001). Post hoc analyses confirmed reductions in T males (*p* = 0.0096) and T females (*p* = 0.0149) compared to sex-matched controls. Notably, no treatment × sex interaction was observed (F(1,20) = 0.02, *p* = 0.8899; Fig. 2).

### Neurostructural changes

**Fig. 3:**
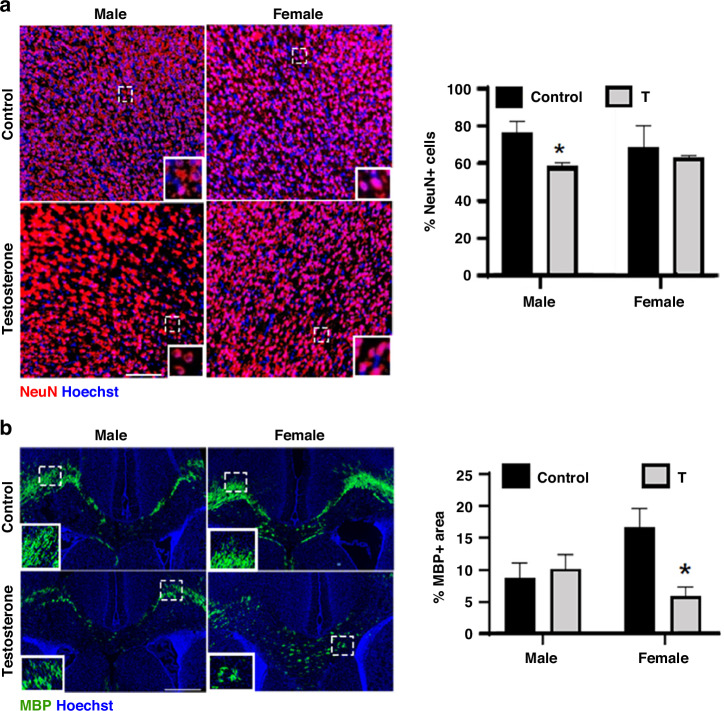
Prenatal T exposure induces sex-specific neurostructural deficits in offspring. **a** Representative immunofluorescent images (scale bar: 50 μm) and quantification of NeuN+ neuronal density in the prefrontal cortex of P9 male and female offspring. Male T-exposed offspring showed reduced NeuN+ cells compared to male controls (*p < 0.05). **b** Representative images (scale bar: 100 μm) and quantification of MBP+ myelinated area in the corpus callosum. Female T-exposed offspring exhibited reduced myelination vs. female controls (*p < 0.05). Insets show a magnified area of the dotted box in the image. Data represent mean ± SD (*n* = 6 litters/group; two-way ANOVA with Tukey’s post hoc test).

We examined neuronal numbers and myelination using NeuN and MBP immunostaining, respectively. Analysis of NeuN immunostaining revealed reduced cortical neuron counts in male T offspring compared to male controls (*p* = 0.0113), but no difference in females (*p* = 0.8771; Fig. 3A). In contrast, MBP immunostaining demonstrated a significant treatment × sex interaction (F(1, 20) = 4.210, *p* = 0.0535), with diminished myelination in the corpus callosum of female T offspring compared to female controls (*p* = 0.0162), but no effect in males (*p* = 0.9732; Fig. 3B).

### Brain DHA deficiency

**Fig. 4:**
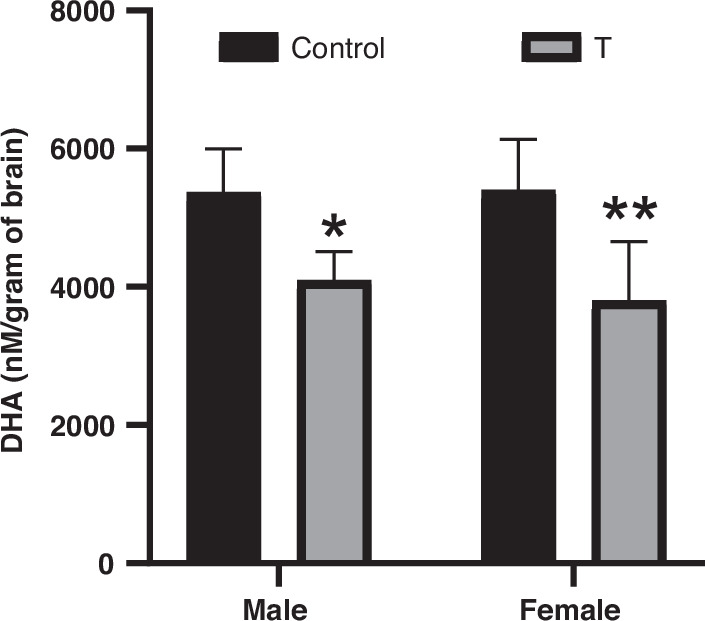
Prenatal T exposure reduces brain DHA concentrations in offspring. Brain DHA levels in P9 male and female offspring. Data represent mean ± SD (*n* = 6 litters/group). *p < 0.05, **p < 0.01 vs. sex-matched controls (two-way ANOVA with Tukey’s post hoc test).

Brain DHA concentrations were significantly reduced in T offspring (main effect of treatment: F(1, 20) = 26.68, *p* < 0.0001). Planned sex-stratified post hoc analyses confirmed deficits in both T males (*p* = 0.0201) and females (*p* = 0.0031) compared to sex-matched controls. Despite these sex-specific differences, no treatment × sex interaction was observed (F(1,20) = 0.3544, *p* = 0.558; Fig. 4).

### Cognitive deficits

#### Spatial working memory

**Fig. 5:**
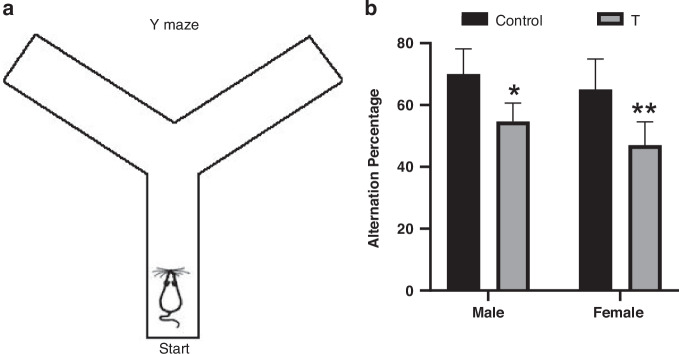
Prenatal T exposure impairs spatial working memory in adolescent offspring. **a** Schematic of the Y-maze apparatus. **b** Spontaneous alternation percentage (10-minute session) in adolescent male and female offspring. Data represent mean ± SD (*n* = 6 litters/group). *p < 0.05, **p < 0.01 vs. sex-matched controls (two-way ANOVA with Tukey’s post hoc test).

Spatial working memory was assessed via spontaneous alternation behavior in the Y-maze (Fig. 5A), a task based on rodents’ innate preference for novel environments.^56,57^ We measured the percentage of successive non-repeating arm entry triplets—a metric requiring transient retention and updating of spatial information.^58^ T offspring exhibited impaired spatial working memory (main effect of treatment: F (1, 20) = 25.93, *p* < 0.001), with planned post hoc tests confirming deficits in both T males (*p* = 0.0168) and T females (*p* = 0.0047) compared to sex-matched controls (Fig. 5B). No treatment × sex interaction was detected (F(1, 20) = 0.1660, *p* = 0.688).

#### Long-term recognition memory

**Fig. 6:**
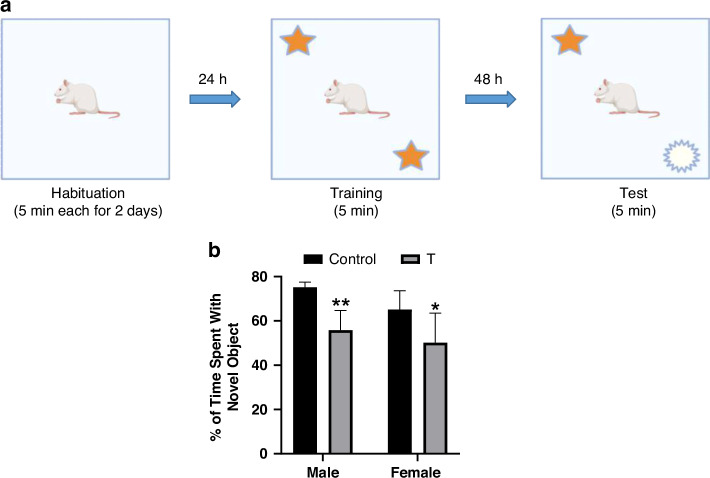
Prenatal T exposure disrupts recognition memory in adolescent offspring. **a** Schematic of the novel object recognition task (10-minute familiarization, 24 h retention). **b** Percentage of time spent exploring the novel object. Data represent mean ± SD (*n* = 6 litters/group). *p < 0.05, **p < 0.01 vs. sex-matched controls (two-way ANOVA with Tukey’s post hoc test).

Long-term recognition memory was evaluated using a novel object recognition task,^59,60^ based on rodents’ natural tendency to explore novel versus familiar objects (Fig. 6A). T offspring exhibited impaired long-term recognition memory (main effect of treatment: F (1, 20) = 21.16, *p* = 0.0002), spending less time exploring novel objects compared to controls. Planned post hoc tests confirmed deficits in both T males (*p* = 0.0077) and T females (*p* = 0.0458) relative to sex-matched controls (Fig. 6B). No treatment × sex interaction was detected (F(1,20) = 0.337, *p* = 0.568).

Analysis of habituation sessions revealed no significant difference in locomotor function between T offspring and controls (main effect of treatment: F(1,20) = 4.14, *p* = 0.055). There was also no significant interaction between treatment and sex (F(1,20) = 0.05, *p* = 0.82). Post hoc comparisons indicated no significant differences in distance traveled for either T males (*p* = 0.724) or T females (*p* = 0.530) relative to their sex-matched controls (Supplemental Table S1).

Similarly, analysis of center time showed no significant differences between T offspring and controls (main effect of treatment: F(1,20) = 2.40, *p* = 0.14), and no significant main effect of sex (F(1,20) = 0.51, *p* = 0.48). There was also no significant interaction between treatment and sex (F(1,20) = 0.51, *p* = 0.48). Post hoc comparisons revealed no significant differences in time spent in the center for either T males (*p* = 0.401) or T females (*p* = 0.934) compared to their respective control groups (Supplemental Table S1).

Analysis of center entries also showed no significant differences between T offspring and controls (main effect of treatment: F(1,20) = 1.94, *p* = 0.18). There was no significant main effect of sex (F(1,20) = 0.84, *p* = 0.37) and no significant interaction between treatment and sex (F(1,20) = 0.15, *p* = 0.71). Post hoc comparisons indicated no significant differences in center entries for either T males (*p* = 0.601) or T females (*p* = 0.889) relative to their sex-matched controls (Supplemental Table S1).

### Social behavioral deficits

Social behavior was assessed in two phases using a three-chamber test.

#### Social approach

**Fig. 7:**
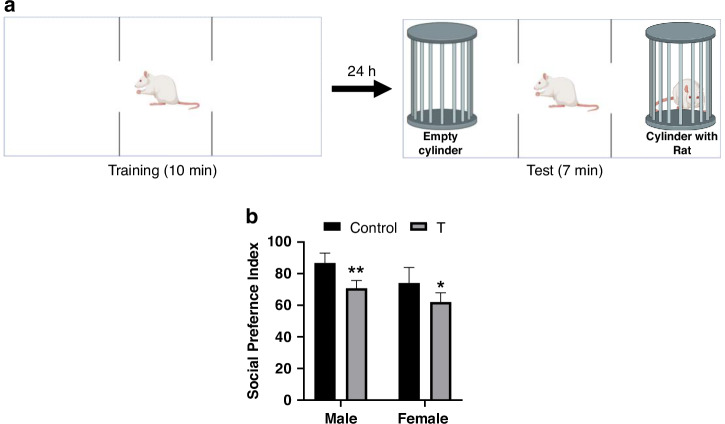
Prenatal T exposure reduces sociability in adolescent offspring. **a** Schematic of the three-chamber social approach task. **b** Social preference index [(time with novel rat − time with empty chamber)/total time × 100]. Data represent mean ± SD (*n* = 6 litters/group). *p < 0.05, **p < 0.01 vs. sex-matched controls (two-way ANOVA with Tukey’s post hoc test).

In the Social Approach phase, a sex- and age-matched unfamiliar rat was placed in one chamber while the other remained empty (Fig. 7A). T offspring exhibited reduced social preference (main effect of treatment: F (1, 20) = 23.93, *p* < 0.001), with deficits in both T males (*p* = 0.0040) and T females (*p* = 0.0354) compared to sex-matched controls (Fig. 7B). A sex effect (F(1,20) = 13.89, *p* = 0.001) indicated higher overall interaction in males, aligning with prior studies [82–84]. No treatment × sex interaction was detected (F(1,20) = 0.489, *p* = 0.493).

#### Social novelty

**Fig. 8:**
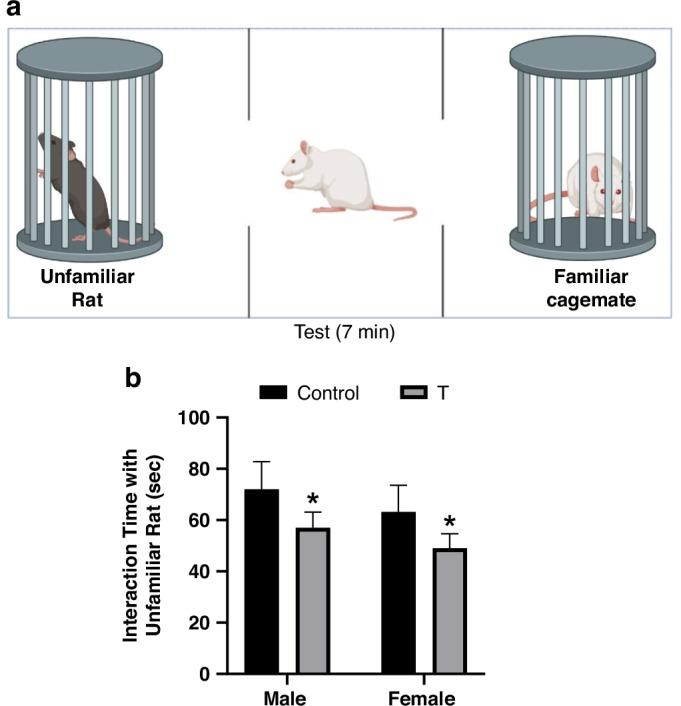
Prenatal T exposure impairs social novelty preference in adolescent offspring. **a** Schematic of the three-chamber social novelty task. **b** Interaction time with an unfamiliar rat. Data represent mean ± SD (*n* = 6 litters/group). *p < 0.05 vs. sex-matched controls (two-way ANOVA with Tukey’s post hoc test).

In the Social Novelty phase, a novel rat was introduced to the previously empty chamber (Fig. 8A) to assess preference for unfamiliar social partners. T offspring displayed diminished preference for novel rats (main effect of treatment: F(1, 20) = 17.14, *p* = 0.0005), with planned post hoc tests confirming reductions in both T males (*p* = 0.0322) and T females (*p* = 0.0456) compared to sex-matched controls (Fig. 8B). No treatment × sex interaction was detected (F(1,20) = 0.014, *p* = 0.907).

## Discussion

Several correlative human studies suggest that elevated maternal T may contribute to psychiatric disorders in offspring, though establishing causal links remains challenging due to the lack of routine prenatal T testing in clinical practice. Nevertheless, emerging evidence—including associations between amniotic fluid T levels and autistic traits^7–9^ and a 66% increased odds of autism in children of mothers with PCOS^10,11^ highlights elevated maternal T as a plausible risk factor. Elevated Maternal T is further implicated in pregnancy complications (e.g., preeclampsia, diabetes) that independently correlate with neurodevelopmental disorders such as ASD.^40–42^ This study provides the first direct evidence in a rodent model that elevated maternal T, mimicking levels associated with these complications, induces neurodevelopmental deficits in offspring. These deficits include sex-specific structural abnormalities (reduced cortical neurons in males, diminished corpus callosum myelination in females) and shared behavioral and biochemical impairments (reduced brain DHA, social deficits, and cognitive dysfunction)—phenotypes that align with core ASD features. These findings extend prior reports of elevated maternal T effects on offspring metabolism and cardiovascular health,^61–63^ providing the first direct evidence of its neural consequences and expanding the recognized spectrum of risks of maternal environmental insults.

Notably, maternal T exposure did not alter gestation length, litter size, or pup survival, indicating specific effects on neurodevelopment rather than general pregnancy outcomes. Birth weights of T-exposed offspring were reduced in both sexes, falling below the 10th percentile of controls—consistent with IUGR in humans.^64^ These results align with clinical reports of maternal T-associated growth restriction^5^ and with previous animal models of prenatal T exposure.^65,66^ In addition to growth deficits, T-exposed pups emitted fewer ultrasonic vocalizations, a measure of early communicative behavior. This deficit mirrors those seen in rodent models of ASD,^67–69^ and parallels diminished social attachment in children with ASD,^70^ highlighting conserved translational relevance.

In addition to communicative impairments, adolescent T-exposed offspring displayed cognitive and social dysfunction, including deficits in spatial working memory (Y-maze alternation) and novelty recognition (novel object test), as well as reduced sociability and social novelty-seeking. These phenotypes recapitulate core ASD-associated traits, such as working memory impairments^71–73^ and aberrant social behavior.^74^ Crucially, unlike models using aromatase inhibitors (e.g., letrozole), which report female-specific deficits,^69^ our study identified neurodevelopmental disruptions in both sexes. This divergence likely stems from distinct hormonal mechanisms: maternal T elevation alters the T-to-estradiol ratio, whereas letrozole suppresses estradiol synthesis. Together, these results align with clinical and preclinical studies demonstrating that prenatal T exposure confers neurodevelopmental risks across sexes,^8,75–77^ supporting a significant role for maternal T in altering offspring social behavior independent of sex.

To address the possibility that motor or anxiety-related confounds influenced our behavioral results, we analyzed the 5-minute habituation sessions from the novel object recognition open field. There were no group differences in total distance traveled, time spent in the center, or number of center entries, indicating that locomotor function was intact and that anxiety-like behavior was not significantly altered in a way that could confound interpretation of our main behavioral outcomes. These findings strengthen the validity of our cognitive and social behavioral assessments. Nevertheless, we acknowledge that more nuanced aspects of anxiety could still influence certain behaviors, and future studies should further explore the potential contribution of anxiety to social and cognitive deficits using dedicated assays.

A central unresolved question is how maternal T disrupts neurodevelopment. While our previous work indicates that this specific maternal T concentration does not directly elevate fetal T levels or alter anogenital distances—suggesting the effects are unlikely due to direct fetal T action^65^ —brain DHA deficiency emerges as a plausible mechanistic pathway. DHA, essential for neurite outgrowth, synaptogenesis,^27,78^ and myelination^79,80^ was significantly reduced in T-exposed offspring. This aligns with studies in rats^43^ and primates^81^ showing placental DHA retention and fetal DHA deficiency under conditions of elevated maternal T. The DHA deficiency in T offspring may drive the observed sex-specific structural deficits: cortical neuron loss in males (where DHA supports neuronal survival) and reduced myelination in females (where DHA is critical for oligodendrocyte function). While this study quantified DHA levels in whole-brain homogenates of P9 offspring, region-specific analysis (e.g., prefrontal cortex, hippocampus) was not feasible due to technical constraints in dissecting neonatal rodent brains. This is an important limitation, as both clinical and preclinical studies indicate that DHA deficiency can exert regionally distinct effects on cortical and hippocampal development, with evidence linking low DHA to neuronal atrophy and impaired synaptic plasticity in these regions.^82–84^ Future studies employing microdissection or advanced imaging approaches will be necessary to clarify whether the observed whole-brain DHA reduction preferentially affects specific neuroanatomical substrates underlying the cognitive and social deficits reported in this study. Although maternal and placental DHA levels were not directly measured in the present cohort, our prior work and recent studies demonstrate that elevated maternal T increases DHA retention within the placenta and reduces fetal serum DHA, without altering maternal DHA levels,^43,85^ indicating impaired placental transfer as a key mechanism. This aligns with findings in pregnancies complicated by preeclampsia or gestational diabetes, where increased placental DHA storage and reduced fetal DHA are observed despite normal maternal status.^85^ Collectively, these data support the model that maternal hyperandrogenism disrupts placental lipid handling, leading to fetal brain DHA deficiency and neurodevelopmental risk. Direct assessment of region-specific brain and placental DHA in future studies will be critical for delineating the mechanistic pathways linking maternal endocrine disruption to offspring brain development. However, the basis for these sex-specific responses remains unclear, and alternative mechanisms, such as oxidative stress or epigenetic modulation of neurodevelopmental pathways, cannot be ruled out and warrant further exploration. Although DHA deficiency is known to impair synaptic plasticity and myelination, the causal relationship between maternal T exposure, DHA deficiency, and neurobehavioral deficits remains unproven. Future studies should directly test whether prenatal or early postnatal DHA supplementation rescues these phenotypes, particularly during critical developmental windows, to clarify mechanistic pathways and therapeutic potential.

We prioritized the frontal cortex (ACC and sensorimotor subregions) and corpus callosum due to their established roles in ASD-relevant behaviors: the ACC regulates social-emotional processing,^86^ while the sensorimotor cortex supports working memory.^87^ These regions exhibit peak developmental sensitivity at P9, when their axonal projections undergo active myelination.^52^ Clinical ASD studies consistently report abnormalities in these circuits, including hypomyelination^88^ and altered connectivity.^89^ Crucially, both regions are highly dependent on DHA for structural integrity—explaining the sex-specific pathologies observed (neuronal loss in males, hypomyelination in females) under conditions of DHA deficiency.^90,91^ While the hippocampus contributes to spatial/recognition memory, its later myelination timeline (>P14)^50^ reduced relevance to our P9 assessment of developmental disruption. Future studies will address hippocampal contributions to long-term memory deficits.

Clinical and preclinical evidence confirms that maternal T elevations (1.5 to 2.4-fold) in preeclampsia and IUGR alter placental structure and androgen receptor signaling.^44,92,93^ While placenta buffers fetal T exposure,^65,93^ T-induced vascular insufficiency and FABP4-mediated lipid sequestration directly impair nutrient transport.^43^ This explains reduced fetal DHA bioavailability despite maternal sufficiency.

These findings advance a critical implication for prenatal care: elevated maternal T represents an underappreciated, modifiable risk factor for offspring neurodevelopmental disorders. Incorporating maternal T screening into prenatal protocols—especially for high-risk pregnancies such as preeclampsia and PCOS—could enable early identification of at-risk dyads. However, implementing routine T measurements will require standardized assays, clear biomarker thresholds (e.g., defining actionable T levels), and careful consideration of ethical and logistical challenges in obstetric practice. A second key implication concerns therapeutic strategies to mitigate neurodevelopmental risk. Our demonstration of shared DHA deficiency in both sexes highlights the promise of nutritional interventions: prenatal or early postnatal DHA supplementation may restore placental-fetal lipid transfer and support neuronal and myelin development. Future clinical trials should test DHA dosing regimens during defined gestational windows and evaluate their efficacy in preventing or ameliorating cognitive and social impairments associated with maternal hyperandrogenism.

Limitations of this work include the inherent challenges of translating rodent social and communicative behaviors to complex human ASD phenotypes. We did not perform formal olfactory testing; however, multiple indirect lines of evidence support intact olfaction during our three-chamber assays. First, all rats displayed robust sniffing (≥2 s bouts directed at stimuli) with no signs of anosmia. Second, deficits in T offspring were consistent across olfaction-independent tasks (ultrasonic vocalizations, Y-maze, novel object recognition). Third, the severity of social impairments paralleled sex-specific neural changes and brain DHA reductions, implicating neurodevelopmental disruption rather than sensory loss. To definitively exclude subtle olfactory deficits, future studies will incorporate formal assays—buried food tests, social odor preference, and habituation–dishabituation. Fourth, although USV and three-chamber tests are validated ASD proxies, they cannot fully capture the disorder’s heterogeneity or its communication-specific deficits in humans, such as nuanced language impairments. Additionally, replicating this finding across experimental models and species is critical to confirm the proposed link. Finally, gestational timing and dose-dependent effects of maternal T on sex-specific outcomes remain unexplored. These variables may clarify why ASD susceptibility differs between sexes and refine predictive biomarkers for high-risk pregnancies.

To dissociate sensory/motor influences from core social-cognitive deficits, future studies should implement a comprehensive neonatal test battery in follow-up studies,^94^ including surface righting reflex, negative geotaxis, cliff aversion, nest-seeking, wire suspension, auditory startle, and eye-opening assessments (P5–P17), complemented by late-infancy elevated plus-maze and novel object recognition tests (P20–P21). These standardized measures will (1) differentiate primary social-cognitive impairments from sensory or motor confounds, (2) map sex-specific developmental trajectories, and (3) identify critical windows during which DHA supplementation or anxiolytic interventions might rescue behavioral and neurostructural deficits.

In conclusion, this study provides experimental evidence that elevated maternal T contributes to neurodevelopmental deficits in offspring, with shared DHA deficiency emerging as a potential mediator. By bridging clinical correlations (e.g., ASD risk in maternal PCOS/preeclampsia) with mechanistic insights from a translational rodent model, these findings underscore two urgent priorities: expanding prenatal screening protocols to identify high-risk pregnancies and prioritizing DHA supplementation trials to mitigate neurodevelopmental risks. Future work should resolve causal relationships (e.g., maternal T → placental DHA retention → neural deficits), explore broader mechanisms (e.g., oxidative stress, epigenetic dysregulation), and define critical windows for intervention to maximize therapeutic efficacy.

## Supplementary information

Supplemental Table S1

## Data Availability

The authors confirm that the data supporting the findings of this study are available within the article.
