# Supplementary material for: Elevated maternal testosterone induces sex-specific neurodevelopmental changes and ASD-related behavioral phenotypes in rat offspring
Source: Pediatr Res. 2025 Oct 1;99(5):1954–63. doi: 10.1038/s41390-025-04425-y (PMC13221296; doi:10.1038/s41390-025-04425-y)
Supplement: Supplementary file 1 — Supplemental Table S1 [file 41390_2025_4425_MOESM1_ESM.pdf]

Supplemental Table S1. Findings from Habituation Analysis

Parameter	Control Males	T Males	Control Females	T Females	Statistical Outcome
Distance (m)	13.1 ± 2.9	12.7 ± 2.4	12.3 ± 2.2	12.0 ± 2.7	Treatment: $F_{1,20}$ = 4.14, p = 0.055; Sex: $F_{1,20}$ = 2.93, p = 0.10; Interaction: $F_{1,20}$ = 0.05, p = 0.82
Center time (s)	97.1 ± 11.8	83.4 ± 17.2	88.7 ± 16.0	83.1 ± 16.8	Treatment: $F_{1,20}$ = 2.40, p = 0.14; Sex: $F_{1,20}$ = 0.51, p = 0.48; Interaction: $F_{1,20}$ = 0.51, p = 0.48
Center entries	14.7 ± 3.6	12.3 ± 3.4	13.0 ± 3.5	11.5 ± 3.6	Treatment: $F_{1,20}$ = 1.94, p = 0.18; Sex: $F_{1,20}$ = 0.84, p = 0.37; Interaction: $F_{1,20}$ = 0.15, p = 0.71

Data presented as mean ± SD; n = 6 litters/sex/group.
NS = Not significant. No post hoc group differences reached statistical significance.
